# Comparison of the LigaSure™ bipolar vessel sealer to monopolar electrocoagulation for thoracoscopic lobectomy and lymphadenectomy: a prospective randomized controlled trial

**DOI:** 10.1007/s00464-023-09892-0

**Published:** 2023-02-15

**Authors:** Piotr Gabryel, Mariusz Kasprzyk, Magdalena Roszak, Alessio Campisi, Szymon Smoliński, Paweł Zieliński, Cezary Piwkowski

**Affiliations:** 1 Department of Thoracic Surgery, Poznan University of Medical Sciences, Szamarzewskiego 62 Street, 60-569 Poznan, Poland; 2 Department of Computer Science and Statistics, Poznan University of Medical Sciences, Poznan, Poland; 3 Department of Thoracic Surgery, University and Hospital Trust–Ospedale Borgo Trento, Verona, Italy

**Keywords:** High-energy device, LigaSure, Lymphadenectomy, Lobectomy, Vessel sealer, Bipolar

## Abstract

**Background:**

High-energy devices allow better vessel sealing compared with monopolar electrocautery and could improve the outcomes of surgical operations. The objective of the study was to compare tissue dissection by the LigaSure™ device with that by monopolar electrocoagulation for thoracoscopic lobectomy and lymphadenectomy.

**Methods:**

This pragmatic, parallel group, prospective randomized controlled trial was funded by the Medtronic External Research Program (ISR-2016–10,756) and registered at www.clinicaltrials.gov (NCT03125798). The study included patients aged 18 years or older, who had undergone thoracoscopic lobectomy with lymphadenectomy at the Department of Thoracic Surgery of Poznan University of Medical Sciences between May 3, 2018, and November 4, 2021. Using simple randomization, the patients were assigned to undergo tissue dissection with either the LigaSure device (study group) or monopolar electrocautery (control group). Participants and care givers, except operating surgeons, were blinded to group assignment. The primary outcome was postoperative chest drainage volume. Secondary outcomes were change of the esophageal temperature during subcarinal lymphadenectomy and C-reactive protein level 72 h after surgery.

**Results:**

Study outcomes were analyzed in 107 patients in each group. We found no differences between the study and control groups in terms of chest drainage volume (550 vs. 600 mL, respectively; *p* = 0.315), changes in esophageal temperature (− 0.1 °C vs. − 0.1 °C, respectively; *p* = 0.784), and C-reactive protein levels (72.8 vs. 70.8 mg/L, respectively; *p* = 0.503). The mean numbers of lymph nodes removed were 12.9 (SD: 3.1; 95% CI, 12.4 to 13.5) in the study group and 11.6 (SD: 3.2; 95% CI, 11.0 to 12.2) in the control group (*p* < 0.001).

**Conclusions:**

The use of the LigaSure device did not allow to decrease the chest drainage volume, local thermal spread, and systemic inflammatory response. The number of lymph nodes removed was higher in patients operated with the LigaSure device, which indicated better quality of lymphadenectomy.

**Supplementary Information:**

The online version contains supplementary material available at 10.1007/s00464-023-09892-0.

The recommended treatment of primary lung cancer is minimally invasive anatomical lung resection and mediastinal lymphadenectomy [1–4]. Postoperative complications occur in 25%–35% of patients and are mostly related to the patients’ age, comorbidities and the extent of surgery [5]. Some complications may result from tissue dissection with the use of electrosurgical devices. For example, extensive lymph node dissection may lead to damage of the esophagus and the bronchi [6], incomplete closure of small vessels may result in postoperative bleeding and chylothorax [7], and thermal spread at the tips of the devices may increase tissue temperature and cause activation of inflammatory response [8].

Monopolar devices have usually been used for tissue dissection and electrocoagulation. Recently introduced advanced bipolar energy device, namely LigaSure™ Sealer/Divider (Medtronic, Minneapolis, MN, USA), has been found to produce stronger vessel seals [9] and was related to decreased thermal spread compared to monopolar devices [10]. Clinical studies in general surgery, urology, and gynecology have shown that in comparison with monopolar devices, the use of the LigaSure device was related to decreased intraoperative blood loss and shorter hospitalization duration [11–13]. A retrospective study in thoracic surgery demonstrated that the use of sealing devices for lobectomy and lymphadenectomy was related to shorter drainage duration and lower incidence of chylothorax [14]. The disadvantage of the LigaSure device is its cost, which significantly exceeds the cost of the monopolar electrocautery. Therefore, to justify its widespread use, LigaSure device should provide clear benefits for the patients. However, so far the evidence for this is relatively weak.

The aim of this study was to compare the short-term results, inflammatory response and local temperature changes caused by the LigaSure device and monopolar electrocautery in patients undergoing VATS lobectomy with lymphadenectomy.

## Materials and methods

This pragmatic, parallel group, randomized controlled trial was approved by the Bioethics Committee of the Poznan University of Medical Sciences, Poznan, Poland (June 16, 2016; number 764/16), and registered at www.clinicaltrials.gov (first posted April 24, 2017; identifier: NCT03125798). The study was funded by the Medtronic External Research Program (ISR-2016–10,756) and was conducted at the Department of Thoracic Surgery, Poznan University of Medical Sciences. Patients were recruited between May 3, 2018, and November 4, 2021.

Eligible were patients admitted for VATS lobectomy and lymphadenectomy for suspected or confirmed primary lung cancer, age of 18 years or older, with the ability to read and understand the information regarding the study, and ability to give informed consent to participate. Exclusion criteria were preoperative radiotherapy or chemotherapy, prior mediastinoscopy or other surgical procedures involving the mediastinum, and ipsilateral chest surgery.

Patients were enrolled in the study by the thoracic surgeons at admission to the department. All patients enrolled gave written informed consent to participate in the study. Using a 1:1 ratio, and a simple randomization technique, we randomly assigned patients to receive one of the two interventions: tissue dissection with the LigaSure device (the study group) or dissection with monopolar device (the control group). The assignment sequence was created with a Web-based random number generator (www.graphpad.com) by the chief investigator. To conceal assignments, we used sequentially numbered, opaque, sealed envelopes that were prepared before the study by an administrative employee of the Department, otherwise not involved in the study, and were opened just before the surgery. From this point, patients’ assignments were known to the operating surgeon. Patients, as well as nurses and doctors engaged in the patients’ perioperative care, including chest tube management and removal, were blinded to the type of intervention.

Operations were performed by one of five board-certified thoracic surgeons experienced in minimally invasive anatomical lung resections. After induction of general anesthesia, a temperature probe was inserted into the patient’s esophagus and positioned at the level of subcarinal nodes, based on the patient's height. The VATS approach consisted of a 4–5 cm-long utility incision in the fourth or fifth intercostal space and usually placement of one or two thoracic trocars. No rib spreaders were used. For the dissection of tissues (including adhesions, the pulmonary ligament, pleura, and all soft tissues surrounding vessels) and for lymphadenectomy, monopolar device was used in the control group and the LigaSure device in the study group. Endostaplers (Endo GIA; Medtronic) were used to divide the pulmonary vessels, bronchi, and interlobar fissures. In cases of conversion to thoracotomy, the incision was enlarged to anterolateral thoracotomy. After the anatomical resection, lymphadenectomy was performed. The lymph nodes removed during lymphadenectomy were counted after the surgery by another thoracic surgeon, who was blinded to patient’s group assignment. At the end of the surgery, one 24-F chest tube was inserted into the pleural cavity and connected to a digital drainage system (Thopaz + ; Medela, Baar, Switzerland). The chest tube was removed after resolution of air leak and when the fluid volume was < 250 mL for 24 h. Perioperative anticoagulation was guided by the Caprini risk assessment model.

The primary outcome measure was total postoperative chest drainage volume, measured from the time the electronic drainage system was turned on at the end of surgery, until the chest tube was removed. Secondary outcome measures were the change in the intraesophageal temperature at the level of the subcarinal lymph nodes (calculated as the difference between the highest temperature during subcarinal nodes dissection and the temperature before subcarinal nodes dissection) and blood C-reactive protein (CRP) levels 72 h after surgery.

We documented demographic characteristics, comorbidities, results of pulmonary function tests, and results of preoperative risk assessment. Data about surgery included approach, type of lobectomy, numbers of lymph nodes and lymph node stations removed, duration of surgery, and estimated blood loss. CRP levels were measured before surgery and 6 and 72 h afterwards. The composition of pleural fluid was evaluated 72 h after surgery for triglyceride levels and hematocrit. The patients were followed up to the 30^th^ postoperative day, or up to the day of hospital discharge if postoperative hospital stay exceeded 30 days.

Clinical outcomes, including complications, and cancer type and stage were documented in accordance with the European Society of Thoracic Surgery/Society of Thoracic Surgeons definitions [15], the World Health Organization classification [16], and the eighth edition of the TNM classification of lung cancer [17], respectively. Assessment of the outcome measures has been performed according to an intention-to-treat design and included patients with conversion to thoracotomy. The study was reported according to the Consolidated Standards of Reporting Trials (CONSORT) recommendations (Supplementary Material).

### Statistical analysis

Sample size was determined with a web-based calculator (https://riskcalc.org/samplesize). This study was powered for superiority of the LigaSure device in accordance with the data for total chest drainage volume in patients after VATS lobectomy and lymphadenectomy with monopolar electrocautery; these data were obtained from the institutional database (2013–2015; *n* = 278). We assumed that if the chest tubes are removed at a drainage volumes lower than 250 mL per day, the reduction of daily amount of chest drainage volume by 125 mL would result in clinically significant reduction in the hospitalization duration of 1 day in half of the patients. If the mean total postoperative chest drainage volume was 750 ± 250 mL, and if the dropout rate were 10%, a sample of 214 patients (107 patients per group) would provide at least 85% power to detect a clinically important difference of 125 mL for this endpoint between the two groups, according to two-sided tests with a 5% level of significance.

The analyzed data were calculated as means ± standard deviations, medians, minimum and maximum values, interquartile ranges (IQRs; quartile 1 to quartile 3), or percentages, as appropriate. We used the Shapiro–Wilk test to check normality of distribution and Levene’s test to check equality of variances. To compare the two unpaired groups, we used the unpaired *t* test for data that followed a normal distribution and had homogeneity of variances; otherwise, we used the Mann–Whitney *U* test. To analyze categorical data, we used the Chi-squared test when the sample size was larger than 40 and all expected values were greater than 5; for other situations, we used Fisher’s exact test or the Chi-squared test with Yates’s correction; or the Fisher-Freeman-Halton test for contingency table larger than 2 × 2 with any expected values was less or equal to 5. All results were considered significant when *p* < 0.05. To perform statistical analyses, we used Statistica 13.0 (StatSoft, Dell, Round Rock, TX, USA) or StatXact 11.0 (Cytel, Cambridge, MA, USA).

## Results

**Fig. 1:**
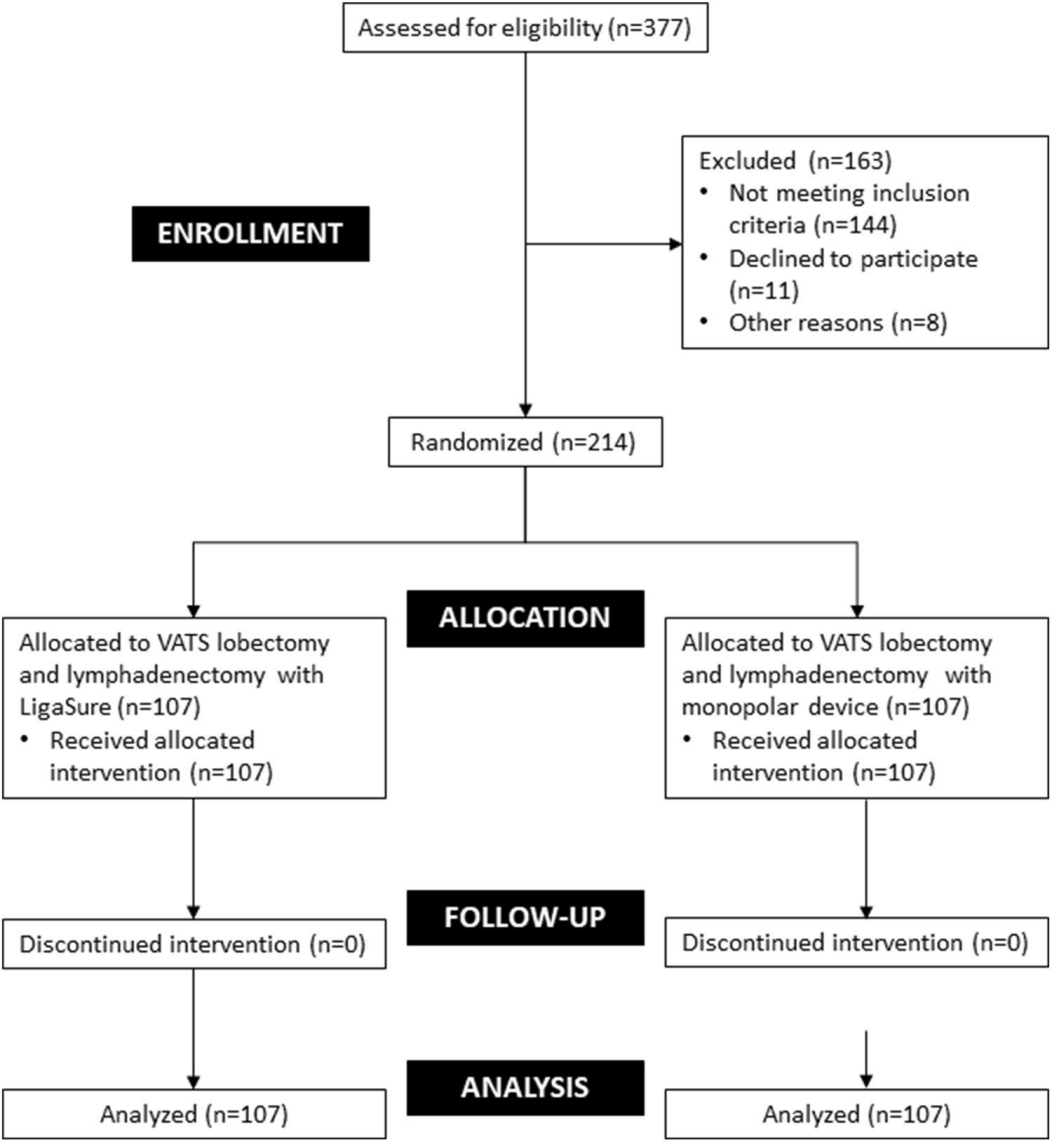
CONSORT flow diagram

Of the 377 patients assessed for eligibility, 214 were randomly assigned to either the study group (LigaSure device) or the control group (monopolar cautery). Primary and secondary outcomes were analyzed in 107 patients in each group (Fig. 1). The baseline and surgical characteristics are listed in Tables 1 and 2, respectively.

**Table 1:** Comparison of baseline characteristics of patients in the study and control groups

Variables	Study group (with LigaSure; *n* = 107)	Control group (with monopolar cautery; *n* = 107)	*p* value
Age (years), mean (SD^a^)	66.0 (7.2)	66.4 (7.5)	0.532
Sex, *n* (%)			0.890
Male	62 (57.9)	63 (58.9)	
Female	45 (42.1)	44 (41.1)	
BMI (kg/m^2^), mean (SD)	26.4 (4.3)	26.7 (5.4)	0.783
ppFEV_1_ (%), mean (SD)	62.7 (16.2)	65.0 (16.9)	0.359
ppDLCO (%), mean (SD)	71.1 (24.7)	73.8 (21.4)	0.325
Current or former smoker, *n* (%)	104 (97.2)	96 (89.7)	0.027^*^
Patients with comorbidities, *n* (%)	86 (80.4)	93 (86.9)	0.196
Chronic obstructive pulmonary disease	36 (33.6)	35 (32.7)	0.885
Coronary arterial disease	16 (14.9)	21 (19.6)	0.366
Cerebrovascular disease	6 (5.6)	45 (4.7)	0.757
Peripheral arterial disease	12 (11.2)	14 (13.1)	0.676
Hypertension	53 (49.5)	59 (55.1)	0.411
Diabetes mellitus	15 (14.0)	29 (27.1)	0.018^*^
Chronic kidney disease	23 (2.8)	2 (1.9)	1.000
Other comorbidity	47 (43.9)	38 (35.5)	0.209
CCI, median (IQR^b^)	3 (2–4)	4 (2–5)	0.088

*BMI* body mass index, *ppFEV*_*1*_* (%)* predicted postoperative percentage of calculated forced expiratory volume in 1 s, *ppDLCO (%)* predicted postoperative percentage of calculated diffusion lung capacity for carbon monoxide, *CCI* Charlson Comorbidity Index

^a^Standard deviation

^b^Interquartile range

^*^Statistically significant with *p* < 0.05

**Table 2:** Comparison of surgical characteristics between study and control groups

Variables	Study group (with LigaSure; *n* = 107)	Control group (with monopolar cautery; *n* = 107)	*p* value
Thoracoscopic approach, *n* (%)			0.422
Multiportal	101 (94.4)	98 (91.6)	
Uniportal	6 (5.6)	9 (8.4)	
Type of lobectomy, *n* (%)			0.761
Right upper	42 (39.3)	37 (34.6)	
Right middle	8 (7.5)	5 (4.7)	
Right lower	12 (11.2)	15 (14.0)	
Left upper	29 (27.1)	33 (30.8)	
Left lower	1416 (15.0)	17 (15.9)	
Operating surgeon, *n* (%)			0.888
Surgeon A	26 (24.3)	29 (27.1)	
Surgeon B	6 (5.6)	6 (5.6)	
Surgeon C	55 (51.4)	50 (46.7)	
Surgeon D	15 (14.0)	14 (13.1)	
Surgon E	5 (4.7)	8 (7.5)	
Conversion to thoracotomy, *n* (%)	7 (6.5)	7 (6.5)	1.000
Reason for conversion to thoracotomy, *n* (%)			0.790
Vascular	4 (57.1)	2 (28.6)	
Anatomy	2 (28.6)	2 (28.6)	
Lymph nodes	1 (14.3)	2 (28.6)	
Technical	0	1 (14.3)	
Number of lymph nodes stations dissected, mean (SD^a^; 95% CI^b^)	5.7 (SD: 1.1; 95% CI, 5.4–5.9)	5.5 (SD: 1.0; 95% CI, 5.3–5.7)	0.233
Number of lymph nodes removed, mean (SD; 95% CI)	12.9 (SD: 3.1; 95% CI, 12.4–13.5)	11.6 (SD: 3.2; 95% CI, 11.0–12.2)	< 0.001^*^
Intraesophageal temperature change during subcarinal lymphadenectomy (℃), median (IQR^c^)	− 0.1 (IQR, − 0.2 to 0.10)	− 0.1 (IQR, − 0.2 to 0.1)	0.784
Duration of surgery (min), mean (SD)	119 (36)	124 (41)	0.366
Median estimated blood loss (mL), median (IQR)	100 (50–150)	100 (50–150)	0.896

^a^Standard deviation

^b^Confidence interval

^c^Interquartile range

^*^Statistically significant with *p* < 0.05

**Fig. 2:**
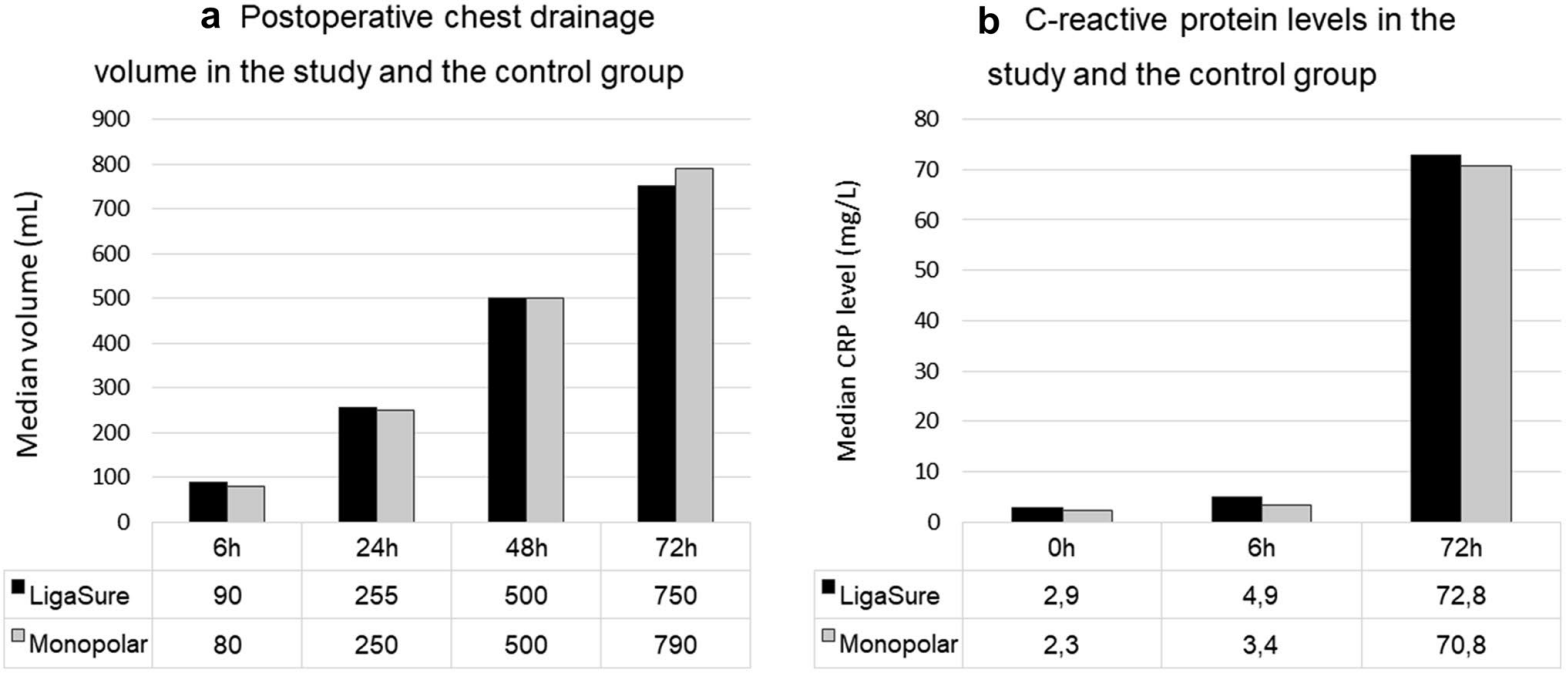
**a** The median chest drainage volumes at 6 h, 24 h, 48 h, and 72 h after surgery. **b** The median C-reactive protein levels before the surgery, and 6 h and 72 h after surgery

The median volume of postoperative chest drainage was 550 mL (IQR, 380–920 mL) in the study group and 600 mL (IQR, 395–960 mL) in the control group; these differences were not significant (*p* = 0.315). Chest drainage volumes 6, 24, 48, and 72 h after surgery also did not differ between the groups (Fig. 2a). The median CRP levels 72 h after surgery were 72.8 mg/L (IQR, 41.5–116.3 mg/L) in the study group and 70.8 mg/L (IQR, 49.6–112.0 mg/L) in the control group (*p* = 0.503). CRP levels before surgery and 6 and 72 h after surgery are plotted in Fig. 2b. Intraesophageal temperature, measured at the level of the subcarinal lymph nodes, dropped in both groups during surgery. The median temperature change during subcarinal lymph nodes dissection was −0.1 °C (IQR, −0.2 to 0.0 °C) in the study group and −0.1 °C (IQR, −0.2 to 0.1 °C) in the control group (*p* = 0.784). The greatest increase in intraesophageal temperature during subcarinal lymphadenectomy was 1.0 °C in the study group and 0.8 °C in the control group. The mean numbers of lymph nodes removed during mediastinal lymph node dissection were 12.9 (SD: 3.1; 95% CI, 12.4 to 13.5) in the study group and 11.6 (SD: 3.2; 95% CI, 11.0 to 12.2) in the control group (*p* < 0.001).

The groups did not differ significantly with regard to estimated intraoperative blood loss, duration of chest tube placement, length of postoperative hospitalization, concentration of triglycerides and hematocrit in the pleural fluid, or complication rate (Tables 2 and 3). Chylothorax developed in 1 patient in the study group and 3 patients in the control group, but the difference in incidence was not statistically significant (*p* = 0.614). There were no postoperative bleeding complications, such as excessive bleeding, or hematoma of the mediastinum, pleural cavity or wounds that required reoperation. None of the patients died during hospitalization or up to 30 days after the surgery.

**Table 3:** Comparison of postoperative characteristics between study and control groups

Variables	Study group (with LigaSure; *n* = 107)	Control group (with monopolar cautery; *n* = 107)	*p* value
Patients with complications, *n* (%)	30 (28.0)	33 (30.1)	0.653
Prolonged air leak	11 (10.3)	16 (15.0)	0.303
Residual air space	8 (7.5)	7 (6.5)	0.789
Atrial fibrillation	9 (8.4)	3 (2.8)	0.075
Chylothorax	1 (0.9)	3 (2.8)	0.614
Atelectasis	3 (2.8)	1 (0.9)	0.614
Pneumonia	3 (2.8)	0	0.245
Recurrent laryngeal nerve palsy	1 (0.9)	0	1.000
Myocardial infarction	0	1 (0.9)	1.000
Other complications	4 (3.7)	6 (5.6)	0.746
Duration of chest drainage (days), median (IQR^a^)	2 (2–4)	3 (2–4)	0.290
Total chest drainage volume (mL), median (IQR)	550 (380–920)	600 (395–960)	0.315
Concentration of triglycerides in pleural fluid 72 h after surgery, (mmol/L), median (IQR)	0.53 (0.39–0.76)	0.58 (0.40–0.80)	0.586
Hematocrit in pleural fluid 72 h after surgery, (mmol/L), median (IQR)	0.01 (0.01–0.02)	0.01 (0.02–0.03)	0.223
Length of hospitalization (days), median (IQR)	6 (4–7)	5 (4–78)	0.362
Readmission, *n* (%)	1 (0.9)	0	1.000

^a^Interquartile range

Nonmalignant histological features were found in three patients in the study group and in five in the control group. The results of postoperative histopathological examinations in patients with a final diagnosis of lung cancer are listed in Table 4.

**Table 4:** Comparison of histopathological characteristics between study and control groups in patients with neoplastic histology

Variables	Study group (with LigaSure; *n* = 104)	Control group (with monopolar cautery; *n* = 102)	*p* value
Histological diagnosis, *n* (%)			0.703
Adenocarcinoma	55 (52.9)	54 (52.9)	
Squamous cell carcinoma	37 (35.6)	32 (31.4)	
Other carcinoma	12 (11.5)	16 (15.7)	
Nodal upstaging (cN0 to pN1 or pN2), *n* (%)	19 (18.3)	18 (17.7)	0.907
Pathological stage, *n* (%)			0.591
I	54 (51.9)	54 (52.9)	
II	37 (35.6)	25 (24.5)	
III	13 (12.5)	23 (22.6)	
pT stage, *n* (%)			0.872
1	34 (32.7)	35 (34.3)	
2	52 (50.0)	45 (44.1)	
3	14 (13.5)	19 (18.6)	
4	4 (3.8)	3 (2.9)	
pN stage, *n* (%)			0.324
0	82 (78.9)	75 (73.5)	
1	13 (12.5)	13 (12.8)	
2	9 (8.7)	14 (13.7)	
Complete resection, *n* (%)	97 (93.3)	91 (89.2)	0.303

## Discussion

We found no differences between the study and control groups in primary and secondary outcome measures, including postoperative chest drainage volume, changes in the esophageal temperature during lymphadenectomy, or CRP levels. The groups also did not differ with regard to complications rates, length of postoperative in-hospital stay and mortality, but the number of lymph nodes removed was higher in the LigaSure group.

The study demonstrated that the LigaSure device and the monopolar device were comparable in terms of the volume of postoperative chest drainage. We also did not find differences in the triglyceride concentrations or values of hematocrit in the pleural fluid, which could be regarded as the laboratory indicators of the quality of the sealing of lymphatic and blood vessels. Previous research in this area has yielded mixed results. One of the findings of a study by Bertolaccini et al. that compared various methods of completion of lung fissures was that the postoperative drainage amount was higher in patients operated with the LigaSure device [18]. On the contrary, a study by Taishi et al. revealed that the use of the LigaSure device for lobectomy and lymphadenectomy was related to decreased intraoperative blood loss and postoperative drainage volume, and shortened postoperative drainage duration [19]. Similar results were obtained by Martucci et al. who demonstrated that the use of the LigaSure device was associated with the decreased cumulative chest tube drainage and reduced duration of mediastinal nodal dissection compared to electrosurgical pencil [20]. The differences in the results of the studies might be explained by the influence of other factors on the postoperative drainage volume. It has been shown that several factors, such as left ventricular ejection fraction, total serum protein level, type of lobectomy and external suction levels were related to the drainage volume [21, 22]. Despite the reported differences, the results of our study indicate that dissection of tissues can be performed effectively with the LigaSure device.

One of the most serious complications of lymphadenectomy are esophageal and bronchial fistulas, which may be a consequence of thermal damage of surrounding tissues during the dissection and coagulation of vessels in the paraesophageal area and under the tracheal bifurcation [23]. An ex vivo study by Sutton et al. and a subsequent studies by Družijanić et al. and Oyama et al. revealed that the temperature at the tips of the devices and the lateral thermal spread during coagulation of the tissues were higher with monopolar diathermy or ultrasonic scissors as with the LigaSure device [10, 24, 25]. This suggests that complications related to thermal damage could occur less frequently in patients in whom LigaSure device is used. Although iatrogenic esophageal thermal injury is exceedingly rare in thoracic surgery, it may be a complication of radiofrequency ablation for atrial fibrillation. As demonstrated by Singh et al., the esophageal temperature monitoring during the ablation could reduce the incidence of esophageal injury [26]. To clinically evaluate the degree of thermal energy dissipation in tissues susceptible to thermal damage, we applied similar methodology and examined the temperature inside the esophagus during dissection of the subcarinal and paraesophageal areas. Changes in intraesophageal temperature did not differ between the two patient groups; also, no patients in either group had significantly higher temperatures (outliers) that could have been caused by spread of thermal energy in the tissues. This suggests that lymphadenectomy can be performed with both the LigaSure device and monopolar coagulation without the risk of thermal damage to the tissues if the devices are used properly.

To assess the role of the energy devices in systemic inflammatory response, we tested the concentration of CRP on the third postoperative day. Previous studies have shown that the CRP level increases in patients after both open and minimally invasive surgery, that levels peak on the third postoperative day, and that the maximum CRP level depends on the extent of the surgical approach: that is, it is lower after VATS than after thoracotomy [27]. In view of Sutton et al.’s findings [10], we hypothesized that the difference in tissue temperature generated by different devices could result in different degrees of surgical trauma, which would be manifested by differences in the maximum CRP level after surgery. Our results showed that although the increase in CRP levels followed a typical pattern in all patients, it was not related to the device used. This indicates that the type of electrosurgical device used for tissue dissection does not influence the systemic inflammatory response.

Important finding of this study was that the number of lymph nodes removed during lymphadenectomy differed significantly between the study and control groups. This difference indicate that the LigaSure device could have improved the quality of lymphadenectomy in comparison with monopolar device. The likely reason for the differences is the insulation of the tips of the instrument and the built-in tissue cutting knife [28]. These properties may facilitate lymphadenectomy in in difficult-to-reach areas, such as aorto-pulmonary window nodes or left subcarinal nodes [29]. As the number of removed lymph nodes has been shown to influence the accuracy of clinical staging and could affect long-term outcomes of surgical treatment of lung cancer, these results may be of importance [30]. Detailed analysis of the issue of relation of the type of electrosurgical device to the quality of lymph node assessment require further studies.

### Limitations

The study has several limitations. First, it was not possible to conceal the type of intervention – dissection with LigaSure versus monopolar device – from the surgeons who performed the operations. Secondly, methodological limitations may have resulted in the non-detection of the differences in intra-esophageal temperature and energy-dissipation-related complications between the groups. Since severe damage to organs directly resulting from the use of electrosurgical devices is a highly improbable complication with a potentially catastrophic impact [31], surgeons must keep extreme caution when using those devices, regardless of the results of our study. Another limitation of the study was that counting the removed lymph nodes might be sensitive to errors that may result from their fragmentation. Weighing the removed tissue might help reduce potential bias but may also have limitations resulting from potential differences in the evaporation of tissue fluid during electrocoagulation. Moreover, although we tried to limit the influence of the human factor on the outcomes, some variations in the quality of surgery related to the differences in the surgeons’ experience, presumably existed. We hope that this pragmatic trial accurately reflected surgical practices in a high-volume thoracic surgery department.

We conclude that the application of the LigaSure device did not reduce chest drainage volume and did not improve short-term outcomes of VATS lobectomy and lymphadenectomy compared to monopolar electrosurgical device. The number of lymph nodes removed during lymphadenectomy was increased with the LigaSure device, which requires evaluation in subsequent studies.

## Supplementary Information

Below is the link to the electronic supplementary material.

Supplementary file1 (DOC 219 kb)
